# Ethylhexylglycerin Impairs Membrane Integrity and Enhances the Lethal Effect of Phenoxyethanol

**DOI:** 10.1371/journal.pone.0165228

**Published:** 2016-10-26

**Authors:** Solveig Langsrud, Katrin Steinhauer, Sonja Lüthje, Klaus Weber, Peter Goroncy-Bermes, Askild L. Holck

**Affiliations:** 1 Nofima—Norwegian Institute of Food, Fisheries and Aquaculture Research, Osloveien 1, N-1430 Aas, Norway; 2 Schülke & Mayr GmbH, Research & Development, Robert-Koch Str. 2, 22851 Norderstedt, Germany; Augusta University Medical College of Georgia, UNITED STATES

## Abstract

Preservatives are added to cosmetics to protect the consumers from infections and prevent product spoilage. The concentration of preservatives should be kept as low as possible and this can be achieved by adding potentiating agents. The aim of the study was to investigate the mechanisms behind potentiation of the bactericidal effect of a commonly used preservative, 2-phenoxyethanol (PE), by the potentiating agent ethylhexylglycerin (EHG). Sub-lethal concentrations of EHG (0.075%) and PE (0.675%) in combination led to rapid killing of *E*. *coli* (> 5 log reduction of cfu after 30 min), leakage of cellular constituents, disruption of the energy metabolism, morphological deformities of cells and condensation of DNA. Used alone, EHG disrupted the membrane integrity even at low concentrations. In conclusion, sub-lethal concentrations of EHG potentiate the effect of PE through damage of the cell membrane integrity. Thus, adding EHG to PE in a 1:9 ratio has a similar effect on membrane damage and bacterial viability as doubling the concentration of PE. This study provides insight about the mechanism of action of a strong potentiating agent, EHG, which is commonly used in cosmetics together with PE.

## Introduction

Survival and growth of microorganisms in cosmetics may lead to product degradation and infection of the end-user [1, 2]. Thus, preservatives, such as 2-phenoxyethanol (PE), organic acids isothiazolinones and parabens are used to maintain the function and ensure that the products are safe to use [3]. However, adverse effects have been described for some preservatives used in various formulations including cosmetics. For example, Lundov *et al*. [4] reported an increase in prevalence of contact allergy to methylisothiazolinone within the last years. It is therefore desirable to keep the concentrations of preservatives as low as possible to reduce exposure of consumers while at the same time maintaining antimicrobial safety of cosmetics. This can be achieved for example by using combinations of preservatives acting synergistically or by adding potentiating agents. The latter are agents which themselves have little lethal or inhibitory effects at the concentrations applied, but enhance the efficiency of other preservatives. For example, the chelator EDTA can potentiate the effect of a range of preservatives, such as quaternary ammonium compounds, parabens and phenolics and is therefore often used in cosmetic formulations [3].

2-Phenoxyethanol is a widely used and well studied compound used for antimicrobial protection of cosmetics [5, 6]. Data from 2010 showed that about 25% of 36 800 cosmetic products sold in USA and 33% of 204 different products in Sweden contained this preservative [5, 7]. According to the cosmetic directive in EU, concentrations of PE up to 1% is allowed for preservative purposes in cosmetics. To obtain sufficient antimicrobial effect it is often combined with other antibacterial agents [8–10]. The mode of action of PE has been investigated extensively, initially by W. Hugo and H. E. Street in the 1950s followed up by P. Gilbert *et al*. in the 1970s [11–17] and was never completely elucidated, but the research revealed that several mechanisms were involved in inhibitory and bactericidal activity. The lethal action of PE on *E*. *coli* was associated with gross membrane damage resulting in leakage of cytoplasmic constituents. It was pointed out that membrane leakage alone could not explain the lethal activity. Experiments at sub-lethal concentrations showed that PE prevented cell growth by inhibiting DNA and RNA biosynthesis [12] as well as the energy metabolism through inhibition of malate dehydrogenase [15] and disruption of the proton gradient [14]. Together the studies showed that PE acted on several targets in the cell depending on the concentrations and most likely cell death was a result of a combination of these mechanisms leading to non-reversible injuries to the cell.

Several investigations have been performed to find synergistic effects between PE and other preservatives. Fitzgerald et al. demonstrated a synergistic effect of PE and chlorhexidine resulting in increased cell death and leakage of potassium and pentose of cells exposed to a combination of the agents compared to each agent separately [18]. Increased leakage could partly explain the synergistic effect. Combining PE with either diazolidinyl urea or methylchloroisothiazolinone/methylisothiazolinone resulted in preservation of cosmetic cream at concentrations below the preservatives’ MIC-values and 10 to 20 times below maximum permitted concentrations. The mechanisms behind the interaction effects were however not studied [19]. PE is often combined with the antifungal preservative chlorphenesin [9]. It has been reported that although PE alone is not skin irritant, even at high concentrations [20], a combination with chlorphenesin results in a synergistic sensory skin irritation [9].

An alternative to building protection against microbial growth by combining different preservatives is to use potentiating agents. There are several advantages of combining preservatives with potentiating agents, for example wider spectrum of activity, enhanced activity at lower concentrations and reduced likelihood of allergic reactions or irritation of the skin for the end user. Ethylhexylglycerin (EHG, 3-[2-(Ethylhexyl)oxyl]-1,2-propandiol), a 1-alkyl glyceryl ether, is a multifunctional additive for cosmetics and is used as a potentiating agent in combination with PE to obtain better protection against microbial growth. A synergistic effect between PE and EHG on the viability of a range of different microorganisms has been reported, both in laboratory tests and in cosmetic products [21]. It has been suggested that the potentiation effect is due to the surfactant properties of EHG, affecting the surface tension properties of bacteria improving the contact between PE and the membrane [21]. Thus, the effect of combination of PE and EHG is well established regarding antimicrobial protection of e.g. cosmetics, but the mechanism of action behind the synergistic effect is not known. A few reports on contact allergy to EHG have been published ([22] and references therein).

The aim of the study was to investigate how ethylhexylglycerin (EHG) potentiates the bactericidal effect of the commonly used preservative 2-phenoxyethanol (PE). In conclusion, EHG damages the cell membrane integrity and together with PE a synergistic action leads to rapid killing of *E*. *coli* associated with leakage of cellular constituents, disruption of the energy metabolism, morphological deformities of cells and condensation of DNA.

## Materials and Methods

The experiments were repeated three times on different days and with freshly prepared cultures and solutions if not stated otherwise.

### Cultivation and preparation of cells

*E*. *coli* ATCC 11229 was inoculated (one colony from Tryptone Soy Agar) in Tryptone Soya Broth (TSB) and incubated over night at 30°C with shaking. Stationary phase cultures were centrifuged (13 000g, 3 min) and the pellets resuspended in sodium phosphate buffer (0.1 mol l^-1^, pH 7.5) if not stated otherwise.

### Antibacterial agents

The antibacterial agents tested were ethylhexylglycerin (EHG, Sensiva SC50), phenoxyethanol (PE) and a mixture of PE (90%) and EHG (10%) (EUX, commercial name Euxyl PE 9010). All agents were provided by Schülke and Mayr, Germany.

### Exposure to antibacterial agents

Cell suspensions (2x10^9^ cfu ml^-1^) were mixed with equal volumes of antibacterial agents (made in double of final concentrations in buffer) or buffer (control). The tubes were incubated at 20°C with shaking. Samples were analysed after 5 min, 30 min, 3 h and 24 h if not otherwise stated.

### Bactericidal activity

After exposure to antibacterial agents, the number of colony forming units was determined by neutralisation in TLSH (3% Tween80, 0.3% Lecithine, 3% Saponine and 0.1% Histidine) and spreading on TSA followed by incubation at 30°C. The detection limit of the test was 10^4^ cfu/ml.

### Lysis

Reduction in optical density at 620 nm was used as a measure of cell lysis after exposure to antibacterial agents. The optical density determined using a spectrophotometer (UV1600PC, VWR, Leuven, Belgium). Lysed cells suspensions were made by sonication (Qsonica, LLC, USA).

### Permeability to dyes

LIVE/DEAD® B*ac*Light^TM^ bacterial Viability Kit (Invitrogen, Oslo, Norway) was used according to the manufacturer’s instructions. The principle of this method is that propidium iodide enters cells with damaged membranes. Dead cells and cells with compromised membranes will fluoresce red. Viable cells will fluoresce green. Fluorescence after exposure to antimicrobial agents was determined by fluorescence microscopy after 3 h and 24 h exposure.

### Leakage of DNA

Cell suspensions were washed three times in PBS, resuspended to 10^9^ cfu ml^-1^ and exposed to antibacterial agents as described above. Bacteria were removed from the solution by centrifugation (13 000g, 3 min) and the amount of DNA in the supernatant measured by absorbance and gel electrophoresis. DNA from a lysed cell suspension was used for measurement of total DNA pool in the cells. The supernatants were extracted with chloroform twice (1:10 v/v) to remove PE, which gives absorption signals at 260 nm. It was confirmed that the extraction procedure resulted in sufficient removal of disturbing antibacterial agents. DNA in the water phase was measured using Nanodrop (Saveen Werner, Sweden), which is a spectrophotometric method for measuring DNA-concentrations (absorbance at 260 nm). It was tested that the instrument gave comparable results with an ordinary spectrophotometer. Agarose gel electrophoresis before and after extraction was used as a confirmatory method for measuring DNA leakage.

### Proton leakage

Initial experiments were performed to determine appropriate volumes of cells and time for acid exposure. The methodology was adapted from Gilbert *et al*. (1977). Over-night cultures were centrifuged (Sorvall, 1500g, 10 min) and washed three times with 2 mol l^-1^ glycylglycine buffer (pH 7.3). The cells were resuspended in buffer to 1x10^11^ cells ml^-1^ and exposed to antimicrobial agents. The buffer in the 5 min control samples was adjusted with 5 mmol l^-1^ HCl to pH 3.8 and the pH monitored after 10 min. The same amount of acid was added to all samples 10 min before the pH measurement (also for the 3 h and 24 h samples). pH was measured before and after addition of HCl and the drop in pH was calculated. For each biological replicate, four control measurements using samples not exposed to antibacterial agents were performed and the order of measurements was varied between each day.

### Inhibition of enzyme activity

Inhibition of malate dehydrogenase activity was determined using a commercial kit (Sigma-Aldrich Chemie GmbH). All reagents (Malate dehydrogenase, Oxaloacetic acid, β-Nicotinamic adenine dinucleotide (reduced form)) were from Sigma-Aldrich Chemie GmbH and the enzymatic assay carried out essentially according to Sigma-Aldrich (http://www.sigmaaldrich.com/technical-documents/protocols/biology/_enzymatic-assay-of-malic-dehydrogenase.html). In short, the substrates oxaloacetic acid and nicotinamide adenine dinucleotide (NADH, reduced form) were mixed in phosphate buffer at room temperature. Different concentrations of preservatives were thoroughly mixed with the reagents. The reaction was started by addition of enzyme and the reduction in absorbance at 340 nm was followed for up to 10 min as NADH was oxidised to NAD^+^. The reaction speed was calculated from slope of the absorbance curves. The different concentrations were tested using three parallels for each biological replicate. Similar results were obtained on two different days.

### Metabolic activity

Inhibition of the metabolic activity was determined using an ATP-luciferin-luciferase-assay (BacTiter-Glo^TM^, Microbial Cell Viability Assay, Promega®, USA). Shortly, the cell suspension was washed twice in PBS and resuspended to 10^7^ cfu ml^-1^ in PBS. The cell suspension was incubated at room temperature for 1 h and aliquots of 50 μl transferred to microtiter plates. Buffers with and without glucose and biocides were added and ATP measured after 5 min, 30 min, 3 h and 24 h incubation.

### Preparation and imaging of samples for electron microscopy

Cells were incubated with biocides for 24 h. After incubation, the cells were centrifuged (2000 g, 1 min) and cell pellets resuspended and fixed in 1 ml 1.25% glutaraldehyde and 2% paraformaldehyde in 0.1 mol l^-1^ Sodium phosphate buffer for 2 h. For scanning electron microscopy (SEM), the fixed cells were washed several times in 0.1 mol l^-1^ PIPES buffer pH 7.2 and dehydrated with 10 min stages in ascending ethanol series (50–100%) or the samples were dehydrated with a gradient series of ethanol (50–100%). The samples were further processed in a BAL-TEC Critical Point Dryer (CPD 030, Germany) and coated with gold/palladium by using a Polaron Sputter Coater (SC 7640, UK). The coated samples were examined and photographed with a Zeiss EVO-50-EP scanning electron microscope at an accelerating voltage of 20 kV in the secondary emission mode. For transmission electron microscopy (TEM), the fixed cells were washed with 0.1 mol l^-1^ sodium cacodylate buffer (SCB), embedded in 3% low melting agarose and post-fixed with 1% osmium tetroxide in 0.1 mol l^-1^ SCB for 1 h. Subsequently, the cells were washed thoroughly in SCB and dehydrated with 10 min stages in ascending ethanol series (50–100%). The cells were embedded in LR White resin (London Resin Company, EMS, England) and ultrathin sections obtained with a LEICA EM UC 6 ultra-microtome. Counterstaining of the sections were performed with 4% aqueous uranyl acetate and 1% potassium permanganate for 10 min. The sections were examined with a FEI MORGAGNI 268 transmission electron microscope operated at 80 kV. Experiments with SEM were repeated four times on different days, while TEM experiments were repeated twice on different days.

### Calculations

Mean values, standard error of the mean and significance testing (t-tests) were calculated using Minitab (Minitab® 17.1.0, www.minitab.com). Cell numbers below the detection limit in the tests were presented as the detection limit.

## Results

**Fig 1:**
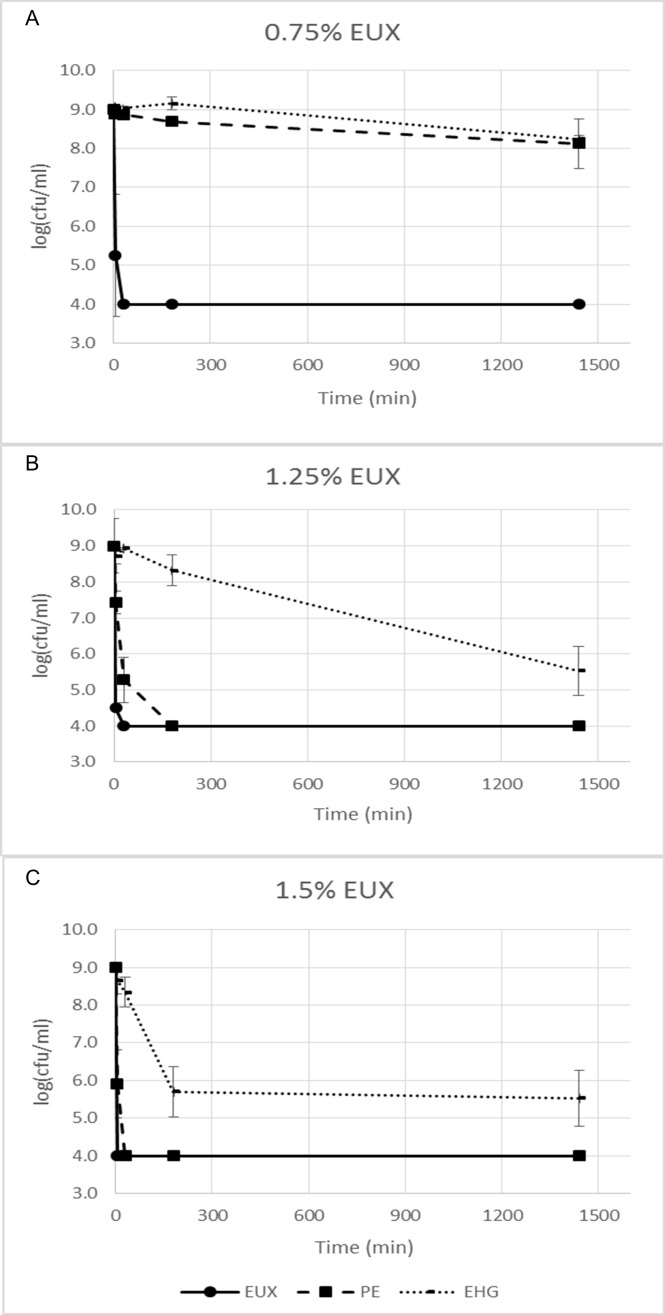
Log_10_ for *E*. *coli* exposed to EUX, PE and EHG. Results for three concentrations of EUX and corresponding concentrations of PE (90% of EUX-conc.) and EHG (10% of EUX-conc.) are shown. (A) 0.75% EUX, (B) 1.25% EUX, (C) 1.5% EUX. Mean values of three replicates and standard error of the mean are shown.

A significant synergy effect of PE and EHG was found as exposure to each of them separately (0.675% PE or 0.075% EHG) did not result in reduction of the cfu of *E*.*coli* after 30 min of exposure, while a combination (EUX 0.75% is a combination of 0.675% PE and 0.075% EHG) resulted in more than 5 log reduction in viability (Fig 1). Higher kill could be obtained by increasing concentration and exposure time for PE and EHG. For a concentration 1.35% PE (30 min exposure time) more than 5 log reduction was obtained by exposure to PE alone. The maximum logarithmic kill found for EHG in these experiments was around 3.5 (0.125% and 0.15% EHG after 24 h exposure). Higher concentrations could not be tested because of low solubility in water.

**Fig 2:**
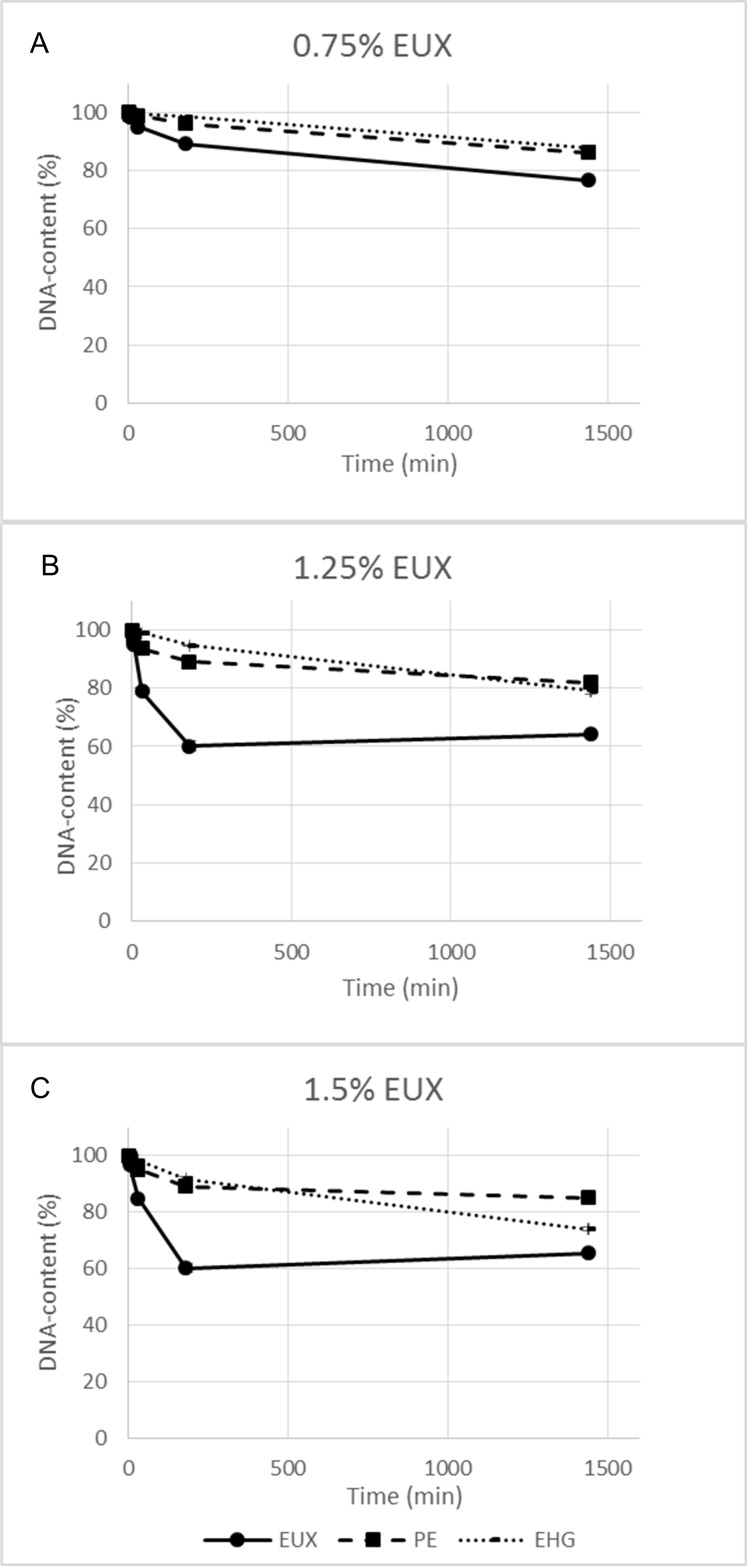
DNA-content of cells exposed to EUX, PE and EHG (% of total DNA pool measured for lysed cells). Results for three concentrations of EUX and corresponding concentrations of PE (90% of EUX-conc.) and EHG (10% of EUC-conc.) are shown. (A) 0.75% EUX, (B) 1.25% EUX, (C) 1.5% EUX. Mean values of three replicates and standard error of the mean are shown.

The permeabilising effect was determined by measuring leakage of DNA (Fig 2), uptake of protons and staining with propidium iodide. Leakage of DNA was not directly correlated to cell death. This can be illustrated by the observation that similar leakage could be found for cells exposed to PE at lethal conditions (e.g. 1.35%, 30 min) as those exposed to EHG without causing death (e.g. 0.15%, 30 min) (Figs 1 and 2). Synergy between PE and EHG on leakage of DNA was observed (Fig 2). For example, exposure to PE (1.35%) and EHG (0.15%) for 3 h separately resulted in a small leakage (about 10%) while a significant synergy effect was observed (p = 0.03) as 40% of the DNA-pool was leaked out after exposure to 1.5% EUX. EHG caused a similar level of DNA-leakage as PE at nine-fold lower concentrations, when comparing corresponding time-concentration points in Fig 2.

Fluorescence microscopy of *E*. *coli* exposed to 0.75% EUX and corresponding concentrations of PE (0.675%) and EHG (0.075%) for 3 h also indicated a similar synergy effect on membrane permeability (results not shown). About 50% of cells exposed to EUX fluoresced red indicating uptake of propidium iodide and thus a compromised membrane. No uptake of propidium iodide could be observed for cells exposed to PE or EHG for 3 h. Interestingly, after 24 h exposure cells exposed to 0.075% EHG appeared equally or more permeable to propidium iodide than those exposed to PE (0.675%) (S1 Fig). For comparison, 80%—> 99% of cells exposed to 0.75% EUX for 24 hours were permeable to PI compared to 10–50% of cells exposed to EHG (0.075%) or PE (0.675%) indicating a lasting synergy effect between PE and EHG (Table E in S1 Dataset).

The lysis followed the same pattern as DNA-leakage. The proportion of intact cells was 60–90% for cell cultures exposed to lethal concentrations (>99.9% reduction in viability) of EUX, PE and EHG. As for DNA-leakage, a synergy effect between PE and EHG was found and exposure to EUX led to rapid lysis at concentrations above 0.75% and a more slow reaction for PE and EHG.

**Fig 3:**
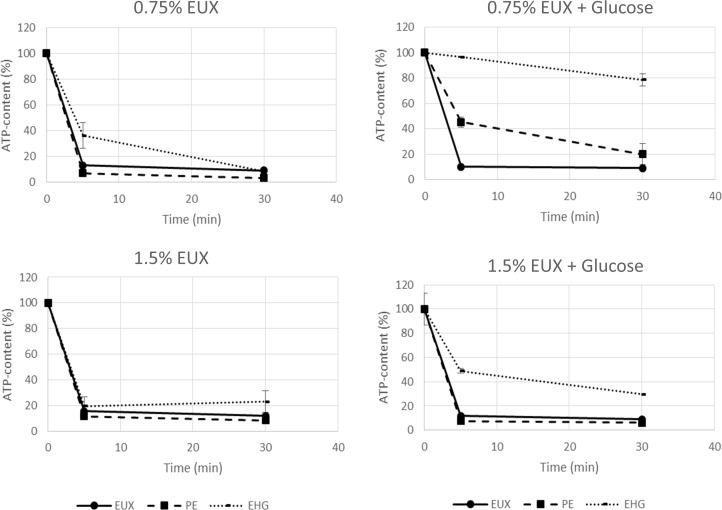
ATP-content of *E*. *coli* exposed to EUX, PE and EHG (% of untreated control cells). (A) and (C): Cells suspended in buffer. (B) and (D): Cells suspended in buffer with glucose. Results for two concentrations of EUX and corresponding concentrations of PE (90% of EUX-conc.) and EHG (10% of EUX-conc.) are shown. (A) and (B) 0.75% EUX, (C) and (D) 1.5% EUX. Mean values of three replicates and standard error of the mean are shown.

Inhibition of metabolic activity was determined by measuring activity of malate dehydrogenase (enzyme in Krebs cycle), ATP-levels (production and use of ATP) and permeability to protons (dissipation of proton motive force). As expected, PE alone and together with EHG partly inhibited malate dehydrogenase. No inhibition was found with EHG alone (concentrations up to 0.12% were tested). Exposure to EUX, PE and EHG resulted in rapid decrease in ATP levels in the absence of glucose (Fig 3). In the presence of glucose, the ATP-content of cells exposed to EHG decreased slowly over time, whereas a more rapid reduction was found for EUX and PE.

**Fig 4:**
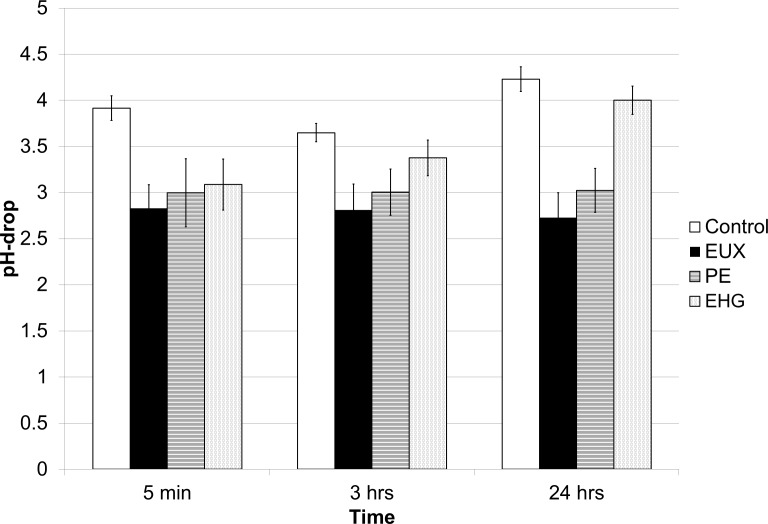
pH-drop of 10^11^ cfu ml^-1^ *E*. *coli* exposed to EUX (0.75%), PE (0.675%) and EHG (0.075%). The cells were exposed for 5 min, 3 h and 24 h and added equal amounts of HCl (determined as the amount needed to reduce the pH of the control after 5 minutes with 4 units). Mean values of three replicates and standard error are shown.

The assay for measuring proton leakage was based on the principle that a smaller pH reduction in cell suspensions treated with an antibacterial agent compared to control cells after adding the same amount of acid will indicate that protons leak into the cells. (Since no carbon source is present in the buffer, the cells will not be able to actively pump out the H^+^.) Thus, a low drop in pH indicates high leakage and a large pH drop indicates less leakage. The drop in pH occurred fast without affecting viability (Fig 4) for EUX, PE and EHG. The degree of leakage remained constant through 24 h for EUX and PE. However, the leakage of protons into EHG-exposed cells decreased over time. One should be aware that the results are not directly comparable to other assays as the cells were not killed by 0.75% EUX because high cell concentrations were used.

**Fig 5:**
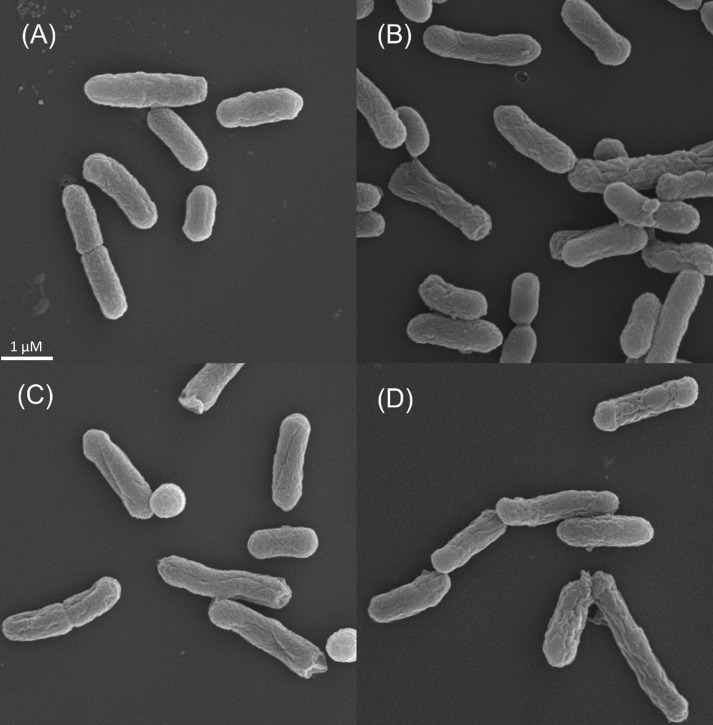
Scanning electron microscopy of cells treated with biocides. (A) Untreated control in Sodium phosphate buffer; (B) 0.75% EUX (0.675% PE + 0.075% EHG); (C) 1.125% PE; (D) 0.125% EHG.

**Fig 6:**
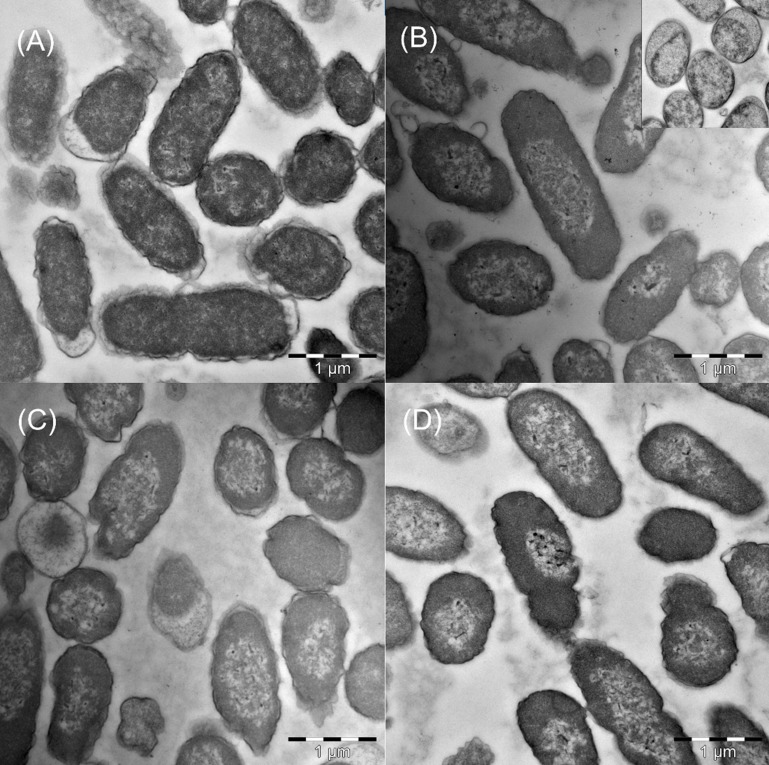
Transmission electron microscopy of cells treated with biocides. (A) Untreated control in Sodium phosphate buffer; (B) 0.75% EUX (0.675% PE + 0.075% EHG); (C) 1.125% PE; (D) 0.125% EHG. Inset in (B) shows leakage of cytoplasmic material out of the cell membrane (not to scale).

Stationary phase cells were treated with biocides, 0.75% EUX (0.675% PE +0.075% EHG), 1.125% PE or 0.125% EHG for 24 h. All these treatments cause > 99.9% reduction in viability. SEM revealed that exposure to these lethal concentrations of EUX, PE and EHG all caused large morphological deformities compared to control cells, with a rougher surface and sometimes with longitudinal furrows (Fig 5). After milder treatments with 0.675% PE and 0.075% EHG, both causing approximately 1 log reduction in viability, variable results were observed with a lesser degree of roughness (not shown). In TEM, control cells showed that the translucent nucleoid material was dispersed throughout the cells (Fig 6). TEM of cells treated with similar high concentrations of biocides 0.75% EUX, 1.125% PE or 0.125% EHG for 24 h revealed condensation of the DNA. In some cases cytoplasmic material appeared to leak out into the space between the cell membrane and the cell wall. This was mainly observed after treatment with 0.75% EUX (inset Fig 6B). Also for TEM intermediate results were observed when cells were treated with 0.675% PE and 0.075% EHG with a lesser degree of condensation of the nucleoid and little leakage of material through the plasma membrane.

## Discussion

Taking advantage of the synergy effect with EHG to keep concentrations low and below the legal limit of PE (1%) seems to be a good approach [21]. The biocidal tests showed rapid kill of bacteria when exposed to a combination of 0.075% EHG and 0.0625% PE but no or little reduction in viability when exposing cells to each compound separately. At least a 5 log reduction in viability could be obtained within 30 min with PE alone by increasing the concentrations (≥1.125%). This synergy effect has also been demonstrated in cosmetic formulations earlier [21].

Although being one of the most commonly used preservatives in cosmetics, and despite the number of studies conducted using a broad approach, the mechanism of action of PE is not completely understood. This study supported earlier findings that PE acts on several targets in the cell, of which some are involved in energy metabolism whereas others in maintaining cell integrity.

Early research by W.B. Hugo suggested that PE affects oxygen uptake by permeabilisation of the membrane without uncoupling activity [16]. However, later Gilbert *et al*. showed that PE disturbs energy metabolism by several mechanisms including dissipation of the proton motive force through proton leakage and inhibition of enzymes involved in Krebs cycle, especially malate dehydrogenase [14, 15]. In accordance with this, the present study also showed rapid proton leakage, reduction in ATP levels and inhibition of malate dehydrogenase after exposure of *E*. *coli* to sublethal levels of PE. Furthermore, as also reported by others [13, 14, 18], increasing concentrations or exposure time resulted in leakage of cellular constituents and lysis of parts of the cell population. It has also been reported that PE affects RNA and DNA synthesis [12]. Gilbert et al [13] observed loss of cytoplasmic material, plasmolysis and large lipophilic blebs when exposing *E*. *coli* to 1% PE. A loss of cytoplasmic material was indicated in the present work, but with leakage out of the cell membrane and not plasmolysis. This may be explained by the use of a lower concentration of PE. In conclusion, as also found by others, the mechanism of action of PE appeared to be broad and it was not possible to point out one single mechanism that correlates with cell death. Thus, as for most biocides several mechanisms are most probably involved, and depend on the concentration applied [23].

In contrast to PE, the mechanism of action of EHG has been much less studied, possibly because the latter shows much less biocidal activity within the in-use range. Sublethal concentrations of EHG rapidly permeabilised the membrane to protons and the ATP-level decreased dramatically in the absence of glucose. Most likely, the proton leakage led to a transient uncoupling of the oxidative phosphorylation from respiration and the proton motive force could not be used to provide energy for ATP synthesis. However, over time, the proton leakage after exposure to EHG decreased, indicating repair. In contrast, a similar recovery was not found after exposure to PE. In the presence of glucose, the ATP-levels were relatively steady over time after exposure to EHG, suggesting that energy metabolism was not permanently altered. After 24 hrs the membrane was apparently disrupted by EHG in such a way that propidium iodide was able to enter the cells despite the observation that the pH-gradient was restored (S1 Fig). Leakage of DNA and lysis appeared to be slower processes and one might speculate that they are consequences rather than causes of cell death.

At time-concentration combinations of EHG resulting in death, leakage of DNA (10%) was observed and electron microscopy showed significant deformities of the cell wall (see also below). The results indicated that EHG acts mainly on the cell membrane and, in contrast to PE, specific actions on energy metabolism, such as inhibition of malate dehydrogenase, are less important. Notably, the same level of leakage was observed at a concentration of EHG that was nine fold lower than that of PE, indicating a higher permeabilising effect of the former. Taking into account the molecular masses and the densities of the compounds give a ratio that is even lower. The results from fluorescence microscopy indicating equal or higher permeability to PI for EHG-treated cells than PE-treated cells supported this hypothesis. Also, the electron microscopy showed similar severe damage of cells after exposure to EHG at lethal concentrations (3 log kill) as for PE (3 log kill) despite EHG concentrations were 10 fold lower. It may be speculated that more gross cell damage is required to obtain the same reduction in viability when specific mechanisms disturbing energy metabolism is not involved. In addition, EHG appeared to condense DNA at lethal levels.

Both SEM and TEM results showed that treatment with lethal concentrations of PE and EHG profoundly influence the cell surface and also acted inside the cell by condensing the bacterial nucleoid. Regarding SEM, a similar crumbling or roughening of the cell walls was observed when *Listeria monocytogenes* were treated with the bacteriocins nisin and pediocin and after a combination treatment of bacteriocins and high hydrostatic pressure (HHP) [24]. Treatment of *Salmonella typhimurium* and *E*. *coli* with HHP gave a similar rough appearance of the cell wall. These treatments all led to a large reduction in viability. The roughening thus seem to indicate severe damage to the cells.

The appearance of the nucleoid in TEM depends heavily on the method used for preparation of the samples [25]. We have employed a method that gives very little condensation of the nucleoid material in healthy cells. The biocide treatment generally resulted in condensed nucleoids. In some cases, however, the nucleoid material became clearly more translucent (clearly lighter than in untreated cells) while still being dispersed over the whole cell. Condensation of the nucleoid has been observed when protein synthesis was inhibited in *E*. *coli* by treating with chloramphenicol [26]. The authors observed DNA condensations with several direct and indirect inhibitors of protein synthesis and also detected condensation with carbonyl cyanide m-chlorophenyl hydrazone (CCCP), an uncoupler of oxidative phosphorylation. They concluded that nucleoid condensation appeared to have resulted from the absence of protein synthesis and also to have occurred under conditions of energy starvation. Several other groups have reported condensation of the bacterial nucleoids by drugs and environmental conditions (see [26] and references therein). It was suggested that inhibition of protein synthesis leads to less interaction of the DNA with the cytoplasm since coupled transcription and translation are interrupted. In this model the nucleoid condensation is a passive consequence of the reduced interaction with the cytoplasm. Both PE and EHG deplete the cells of energy, which reduce protein synthesis and could lead to condensation, as was the case for CCCP. PE is reported to completely inhibit both DNA, RNA and protein synthesis [12]. PE is also reported to precipitate nucleic acids and proteins at higher concentrations [18]. It is still an open question if inhibition of protein synthesis is part of the killing mechanism when treating the cells with PE or EHG and if the condensation of DNA is directly involved in reduction of viability of the cells or a consequence of reduced protein synthesis.

Gilbert et al [13] observed plasmolysis and extracellular lipophilic globules in TEM when *E*. *coli* was treated with 0.5% PE for 15 min. We did not observe such globules in our preparations. The significance of the apparent leakage of cytoplasmic proteins out of the cell membrane is difficult to evaluate, since it was observed in only few cells.

A pronounced synergy effect between PE and EHG was observed as the combination resulted in rapid cell death at concentrations and exposure times which gave insignificant reduction in viability when the cells were exposed to each agent alone. Adding EHG to PE in a 1:9 ratio had a similar effect on membrane damage and bacterial viability as a two-fold higher conc of PE alone. Potentiation of the lethal effect of PE by EHG for *Pseudomonas aeruginosa* and *Aspergillus niger* has been observed earlier [21] and it was postulated that EHG, as a surface active agent, enabled better contact between PE and targets on the cell membrane. In the present work, the synergy effect was apparent when comparing leakage of DNA, permeabilisation to propidium iodide and general membrane damage as observed by SEM. No synergy between EHG and PE was found with regard to proton leakage (Fig 4). Apparently, the mechanisms involved when exposing cells to EUX were similar to those involved when exposing *E*. *coli* to higher concentrations of PE, but the action was faster and at a lower concentration. Possibly EHG potentiate PE through permeabilisation of the membrane leading to more severe damage and causing increased cell death. The kinetics may also play an important role, as it is more difficult for the cells to adapt to substances acting fast. The results support that it is important to include a range of exposure times and concentrations in investigations of antibacterial compounds with a broad range of mechanisms of action.

As also reflected in the present study, the mechanism of action of preservatives and other biocides is often complex, with a wide range of targets. Different mechanisms may be involved depending on experimental conditions. Also, the reversibility of the injuries inflicted may depend on both the type and degree of damage, and in addition on the conditions for recovery [23, 27]. These factors complicate mechanistic studies and one should be aware of this complexity when using different assays for measuring viability. For example, according to the manufacturers of LIVE/DEAD® *Bac*Lght^TM^ Bacterial Viability Kit and BacTiter-Glo^TM^ Microbial Cell Viability Assay, the assays can be used to measure cell viability. For antibacterial agents that acts on several targets in the cells, a good correlation between e.g. propidium iodide permability or ATP-content and cell death cannot be expected. This can be illustrated by EHG permeabilising cells to propidium iodide and reduce ATP-levels at concentrations not causing death. These assays are, however, valuable for investigation of mechanism of action of different substances.

The present study demonstrated that sub-lethal concentrations of EHG disrupt the membrane integrity and that combination with sub-lethal concentrations of PE results in rapid killing (> five log reduction after 30 min). This combination is associated with leakage of cell constituents, disruption of energy metabolism, morphological deformities and DNA condensation. To obtain reductions in the use of preservatives in cosmetics, taking advantage of multifunctional activities of cosmetic ingredients resulting in synergy effects with a preservative, is obviously a good alternative to combining different preservatives.

## Supporting Information

S1 Dataset **Table A:** Raw data for Fig 1.**Table B:** Raw data for Fig 2. **Table C:** Raw data for Fig 3. **Table D:** Raw data for Fig 4. **Table E:** Raw data for calculating percentages of *E*.*coli* permeable to LIVE/DEAD staining
(XLSX)

S1 Fig Fluorescence microscopy of *E*. *coli* exposed to 0.75% EUX, 0.675% PE and 0.075% EHG for 24 h. Green: SYTO 9 stain, Red: propidium iodide stain.
(TIF)

## References

[1] Alvarez-Lerma F, Maull E, Terradas R, Segura C, Planells I, Coll P, et al Moisturizing body milk as a reservoir of Burkholderia cepacia: outbreak of nosocomial infection in a multidisciplinary intensive care unit. Crit Care. 2008;12(1):R10 (doi:.1186/cc6778). 10.1186/cc6778 18237375 PMC2374635

[2] Martin M, Winterfeld I, Kramme E, Ewert I, Sedemund-Adib B, Mattner F. Outbreak of Burkholderia cepacia complex caused by contaminated alcohol-free mouthwash. Anaethesist. 2012;61:25–9. 10.1007/s00101-011-1954-4 22273822

[3] Hiom SJ. Preservation of medicines and cosmetics In: Fraise AP, Lambert PA, Maillard JY, editors. Disinfection, Preservation and Sterilization. 4th ed. ed. Oxford, UK: Blackwell Publishing; 2004.

[4] Lundov MD, Opstrup MS, Johansen JD. Methylisothiazolinone contact allergy—growing epidemic. Cont Dermat. 2013;69:271–5. 10.1111/cod.12149 24117738

[5] Yazar K, Johnsson S, Lind ML, Boman A, Liden C. Preservatives and fragrances in selected consumer-available cosmetics and detergents. Cont Dermat. 2011;64(5):265–72. 10.1111/j.1600-0536.2010.01828.x 21138445

[6] Steinberg DC. Voluntary registration of cosmetics and 2007 frequency of preservative use. Cosmet Toil. 2008;123:47–52.

[7] Steinberg DC. 2010 Frequency of preservative use. Cosmet Toil. 2010;125 (11):46–51.

[8] Regulation (EC) No. 1223/2009 of the European Parliament and of the Council of 30 November 2009 on cosmetic products (recast), (2009).

[9] Lee E, An SS, Choi DW, Moon S, Chang IS. Comparison of objective and sensory skin irritations of several cosmetic preservatives. Cont Dermat. 2007;56(3):131–6. 10.1111/j.1600-0536.2007.01001.x 17295686

[10] Krowka J, Loretz L, Brzuska K, Almeida J, Diehl M, Gonsior S, et al Phenoxyethanol as a Safe and Important Preservative in Personal Care. Cosm & Toil. 2014;129:24–7.

[11] Gilbert P, Beveridge G, Crone PB. Action of phenoxyethanol upon respiration and dehydrogenase enzyme-systems in *Escherichia-coli*. J Pharm Pharmacol. 1976;28:P51-P. 12328

[12] Gilbert P, Beveridge EG, Crone PB. Effect of 2-phenoxyethanol upon RNA, DNA and protein-biosynthesis in *Escherichia-coli* NCTC 5933. Microbios. 1980;28(111):7–17. 6161294

[13] Gilbert P, Beveridge EG, Crone PB. Lethal action of 2-phenoxyethanol and its analogs upon *Escherichia-coli* NCTC 5933. Microbios. 1977;19(76):125–41. 366338

[14] Gilbert P, Beveridge EG, Crone PB. Effect of phenoxyethanol on permeability of *Escherichia-coli* NCTC 5933 to inorganic-ions. Microbios. 1977;19(75):17–26. 357929

[15] Gilbert P, Beveridge EG, Crone PB. Inhibition of some respiration and dehydrogenase enzyme-systems in *Escherichia-coli* NCTC 5933 by phenoxyethanol. Microbios. 1977;20(79):29–37. 362131

[16] Hugo WB. The Action of Phenol and 2-Phenoxyethanol on the Oxidation of Various Substances by *Escherichia coli* and by a Disrupted Cell Preparation of the Organism. J Gen Microbiol. 1956;15(2):315–23. 10.1099/00221287-15-2-315 13376873

[17] Hugo WB, Street HE. The Effect of Phenol, 2-Phenoxyethanol and Cetyltrimethylammonium Bromide on the Oxidation of Various Substrates by *Escherichia coli*. J Gen Microbiol. 1952;6(1–2):90–4. 10.1099/00221287-6-1-2-90 14927855

[18] Fitzgerald KA, Davies A, Russell AD. Mechanism of action of chlorhexidine diacetate and phenoxyethanol singly and in combination against gram-negative bacteria. Microbios. 1992;70(284–85):215–30. 1406341

[19] Lundov MD, Johansen JD, Zachariae C, Moesby L. Low-level efficacy of cosmetic preservatives. Int J Cosmetic Sci. 2011;33(2):190–6. 10.1111/j.1468-2494.2010.00619.x 21272037

[20] Scognamiglio J, Jones L, Letizia CS, Api AM. Fragrance material review on 2-phenoxyethanol. Food Chem Toxicol. 2012;50:S244–S55. 10.1016/j.fct.2011.10.030 22036980

[21] Beilfuss W, Leschke M, Weber K. A new consept to boost the preservative efficacy of phenoxyethanol. SÖFW Journal. 2005;11(131):2–7.

[22] Harries C., Mühlenbein S., Geier J. and Pfützner W. Allergic contact dermatitis caused by ethylhexylglycerin in both an ointment and a skin aerosol. Cont Dermat. 2016; 74: 181–182. 10.1111/cod.12471 26899807

[23] Denyer SP. Mechanisms of action of antibacterial biocides. Int Biodeter Biodegrad. 1995;36(3–4):227–45.

[24] Kalchayanand N, Dunne P, Sikes A, Ray B. Viability loss and morphology change of foodborne pathogens following exposure to hydrostatic pressures in the presence and absence of bacteriocins. Int J Food Microbiol. 2004;91(1):91–8. 10.1016/S0168-1605(03)00324-6 14967564

[25] Robinow C, Kellenberger E. The Bacterial Nucleoid Revisited. Microbiol Rev. 1994;58(2):211–32. 7521510 10.1128/mr.58.2.211-232.1994 PMC372962

[26] Zusman DR, Carbonel A, Haga JY. Nucleoid Condensation and Cell-Division in *Escherichia coli* Mx74t2 Ts52 after Inhibition of Protein-Synthesis. J Bacteriol. 1973;115(3):1167–78. 4580561 10.1128/jb.115.3.1167-1178.1973 PMC246367

[27] Maillard JY. Bacterial target sites for biocide action. J Appl Microbiol. 2002;92:16s–27s. 12000609

