# Supplementary figures and images for: Ethylhexylglycerin Impairs Membrane Integrity and Enhances the Lethal Effect of Phenoxyethanol
Source: PLoS One. 2016 Oct 26;11(10):e0165228. doi: 10.1371/journal.pone.0165228 (PMC5082626; doi:10.1371/journal.pone.0165228)

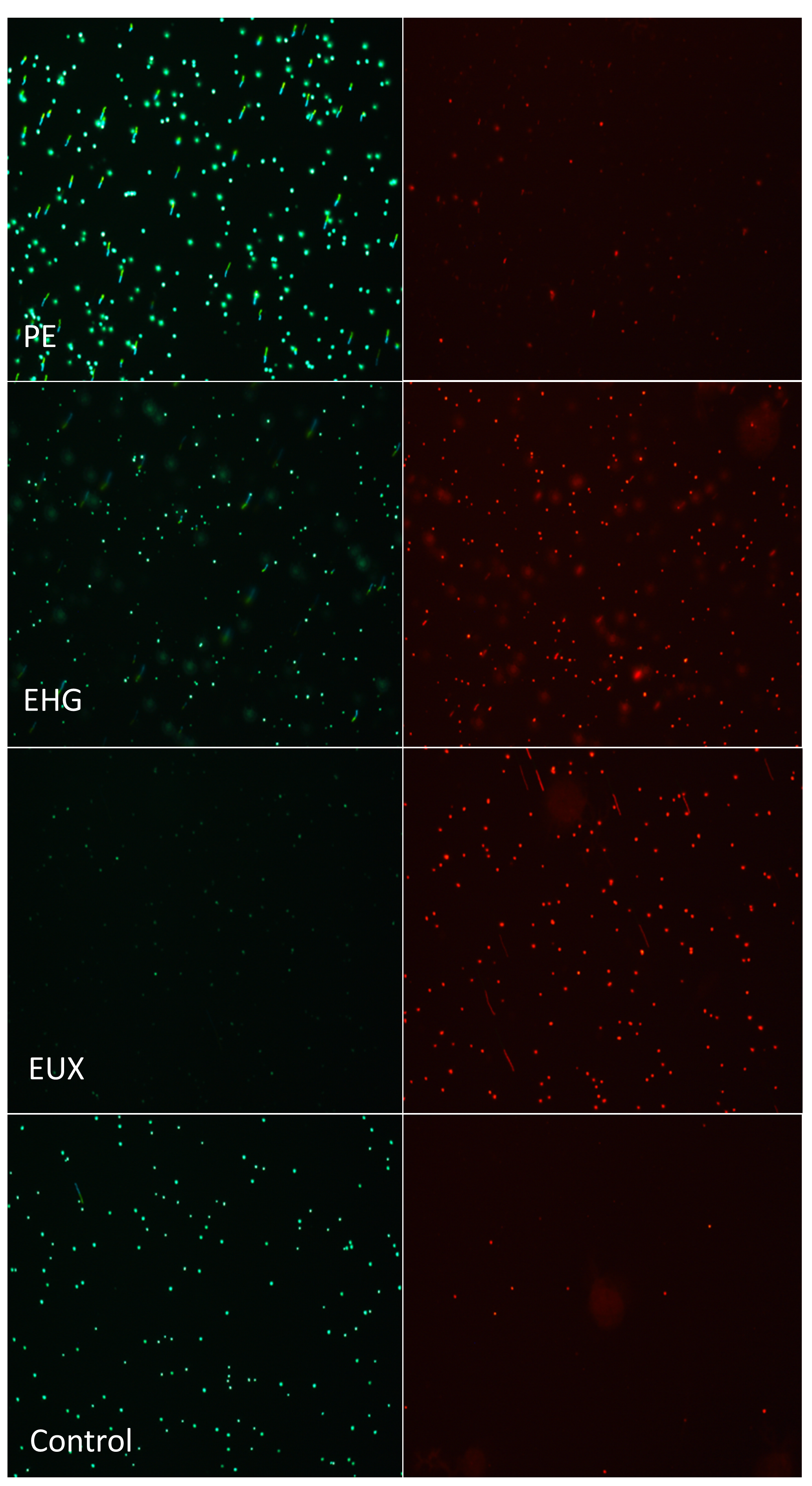

Supplement: S1 Fig — Green: SYTO 9 stain, Red: propidium iodide stain. (TIF) [file pone.0165228.s002.tif]
